# Cryptic population structure and transmission dynamics uncovered for *Schistosoma mansoni* populations by genetic analyses

**DOI:** 10.1038/s41598-022-04776-0

**Published:** 2022-01-20

**Authors:** Jeffrey C. Long, Sarah E. Taylor, Lucio M. Barbosa, Luciano K. Silva, Mitermayer G. Reis, Ronald E. Blanton

**Affiliations:** 1 Department of Anthropology, University of New Mexico, Albuquerque, NM 87131 USA; 2 Bahiana School of Medicine and Public Health, Av. Silveira Martins, n 3386, Salvador, Bahia 41150-100 Brazil; 3 Gonçalo Moniz Institute, Oswaldo Cruz Foundation, Ministry of Health, Waldemar Falcão Street, 121, Salvador, Bahia Brazil; 4 Faculty of Medicine of Bahia, Federal University of Bahia, Praça Conselheiro Almeida Couto, s/n - Largo do Terreiro de Jesus, Salvador, BA 40025-010 Brazil; 5 Department of Epidemiology of Microbial Diseases, Yale School of Public Health, Yale University, 60 College Street, P.O. Box 208034, New Haven, CT USA; 6 Department of Tropical Medicine, School of Public Health and Tropical Medicine, Tulane University, 1440 Canal Street, Suite 2306, New Orleans, LA 70112 USA

**Keywords:** Genetics, Genetic markers, Population genetics, Diseases, Infectious diseases, Parasitic infection

## Abstract

Patterns of diversity in pathogen genomes provide a window into the spatiotemporal spread of disease. In this study, we tested the hypothesis that *Schistosoma mansoni* parasites form genetic clusters that coincide with the communities of their human hosts. We also looked for genetic clustering of parasites at the sub-community level. Our data consists of 14 microsatellite DNA markers, typed from pooled DNA samples from $N=254$ infected individuals living in three Brazilian communities. We found a one-to-one correspondence between genetic clusters found by K-means cluster analysis and communities when $K = 3$. These clusters are also easily identified in a neighbor-joining tree and principal coordinates plots. K-means analysis with $K > 3$ also reveals genetic clusters of parasites at the sub-community level. These sub-clusters also appear on the neighbor-joining tree and principal coordinates plots. A surprising finding is a genetic relationship between subgroups in widely separated human communities. This connection suggests the existence of common transmission sites that have wide influence. In summary, the genetic structure of *S. mansoni* in Brazil juxtaposes local isolation that is occasionally broken by long-range migration. Permanent eradication of schistosomes will require both local efforts and the identification of regional infection reservoirs.

## Introduction

Schistosomiasis is an important cause of mortality globally that may account for up to 200,000 deaths annually^1^. *Schistosoma mansoni* is one of six species of parasitic trematode worms that cause the disease in humans. This species infects millions of people throughout sub-Saharan Africa, the Arabian Peninsula, Madagascar, the Caribbean, and South America^2^. Population genetic analysis of whole genome sequences indicates that *S. mansoni* emerged in Africa as a distinct species approximately 126.5 thousand years ago when it split from a common ancestor with its congener *S. rodhaini*. *Schistosoma mansoni* was dispersed to the Americas within the last five centuries by the trans-Atlantic Slave Trade^3^.

The *S. mansoni* life cycle requires two hosts, humans, where sexual reproduction takes place, and freshwater snails in the genus *Biomphalaria spp*., where the parasite reproduces asexually. Since the parasite is long-lived in the human host and the snail life span is only about three months, most of the parasite’s genetic potential resides in the human population. Adult worms live and reproduce in male-female pairs in the mesenteric veins that drain the host’s intestines. Female worms release fertilized eggs into the host’s blood stream until they lodge in tissues or pass through the intestinal wall and are expelled in feces^4^. Upon reaching fresh water, eggs hatch and release miracidia that infect the snail intermediate host, multiply asexually, and mature into the cercarial stage which leaves the snail. The cercariae penetrate human skin when contact is made with contaminated water^4^. Schistosomiasis is most common in rural communities. However, migration of people from the countryside to cities has resulted in presence and transmission of schistosomaisis in urban environments^5,6^.

Population genetic analyses can provide insight into transmission patterns and responses by schistosome populations to control programs^7–9^. *Schistosoma mansoni* has a multitiered population structure. The convention among schistosome researchers is to refer to the worms inhabiting a single person as an “infrapopulation”^10^. The number of worms in an infrapopulation is difficult to measure. An autopsy study of 96 people found an average of 45.5 pairs per person with a strong positive skew^11^. Recent studies employing indirect methods produced similar, albeit somewhat higher, estimates of infrapopulation sizes^12,13^. The next level of population structure is the “component population” which is the collection of infrapopulations in a given geographic location^10^. Gower and colleagues conducted two studies of *S. mansoni* population genetic structure in Africa^7,14^. Both studies document high diversity within and between infrapopulations within component populations. One study on pan-African populations found low differentiation between children in different schools within the same country, and substantial differentiation between infrapopulations in school children living in different countries^14^. The other study found low differentiation between nearby local schools in Kenya but high differentiation when comparing the same schools before and after mass praziquantel treatment^7^. A study of *S. mansoni* in four geographically separated communities in Ethiopia^12^ found moderately high differentiation among communities. A study of *S. mansoni* in Brazil found significant differentiation between schistosomes in two rural communities, although these communities are separated by only 8 km and connected by a highway. An analysis of measurable epidemiological variables in the Brazilian study’s participants did not reveal factors that contribute to isolation or connections between the schistosome populations^15^. In summary, schistosome transmission dynamics are likely to be highly influenced by local factors and to differ by place.

In the present study, we perform a detailed analysis of patterns of genetic differentiation and relatedness in *S. mansoni* carried by infected residents of three communities located in the State of Bahia, Brazil. The first community is the urban neighborhood of São Bartolomeu, which is located in the City of Salvador. The second and third communities, Jenipapo and Volta do Rio, are rural communities located 200 km southwest from Salvador in the municipality of Ubara. We analyze allele frequency profiles for a panel of 14 microsatellite loci for each infrapopulation. The allele frequency profiles were obtained from assays of pooled DNA samples according to previously published methods^16^. We begin by computing Nei’s hierarchical gene differentiation statistics for infrapopulations and communities^17,18^. However, the genetic structure of *S. mansoni* in Brazil is complicated, perhaps by repeated founder effects caused by its recent introduction and range expansion. We augment the simple gene differentiation statistics using cluster analyses and principal coordinates.

**Table 1:** Genetic loci and characteristics.

Locus	Alleles	$J_S$	$J_C$	$J_T$	$G_{SC}$	$G_{CT}$	$G_{ST}$
SMMS2	4	0.63	0.62	0.62	0.027	0.000	0.026
SMMS13	5	0.696	0.678	0.677	0.056	0.004	0.06
SMMS16	8	0.245	0.23	0.207	0.019	0.029	0.048
SMMS3	13	0.169	0.152	0.147	0.021	0.005	0.026
SMMS18	15	0.251	0.226	0.203	0.033	0.029	0.061
SMMS21	9	0.557	0.542	0.376	0.034	0.266	0.291
SMDA23	12	0.308	0.286	0.286	0.031	0.000	0.032
1F8A	11	0.287	0.265	0.251	0.029	0.019	0.048
13TAGA	11	0.288	0.272	0.253	0.022	0.025	0.047
SM13-410	8	0.45	0.433	0.296	0.029	0.194	0.218
SMU31768	14	0.200	0.193	0.19	0.008	0.003	0.012
15J15A	10	0.275	0.263	0.261	0.016	0.003	0.019
29E6A	9	0.281	0.259	0.25	0.03	0.012	0.041
SM13-478	13	0.286	0.209	0.204	0.097	0.006	0.103
Multiple Locus	–	0.352	0.331	0.302	0.032	0.041	0.072
Lower 95%	–	0.275	0.255	0.231	0.022	0.010	0.038
Upper 95%	–	0.443	0.424	0.394	0.046	0.086	0.116

**Figure 1:**
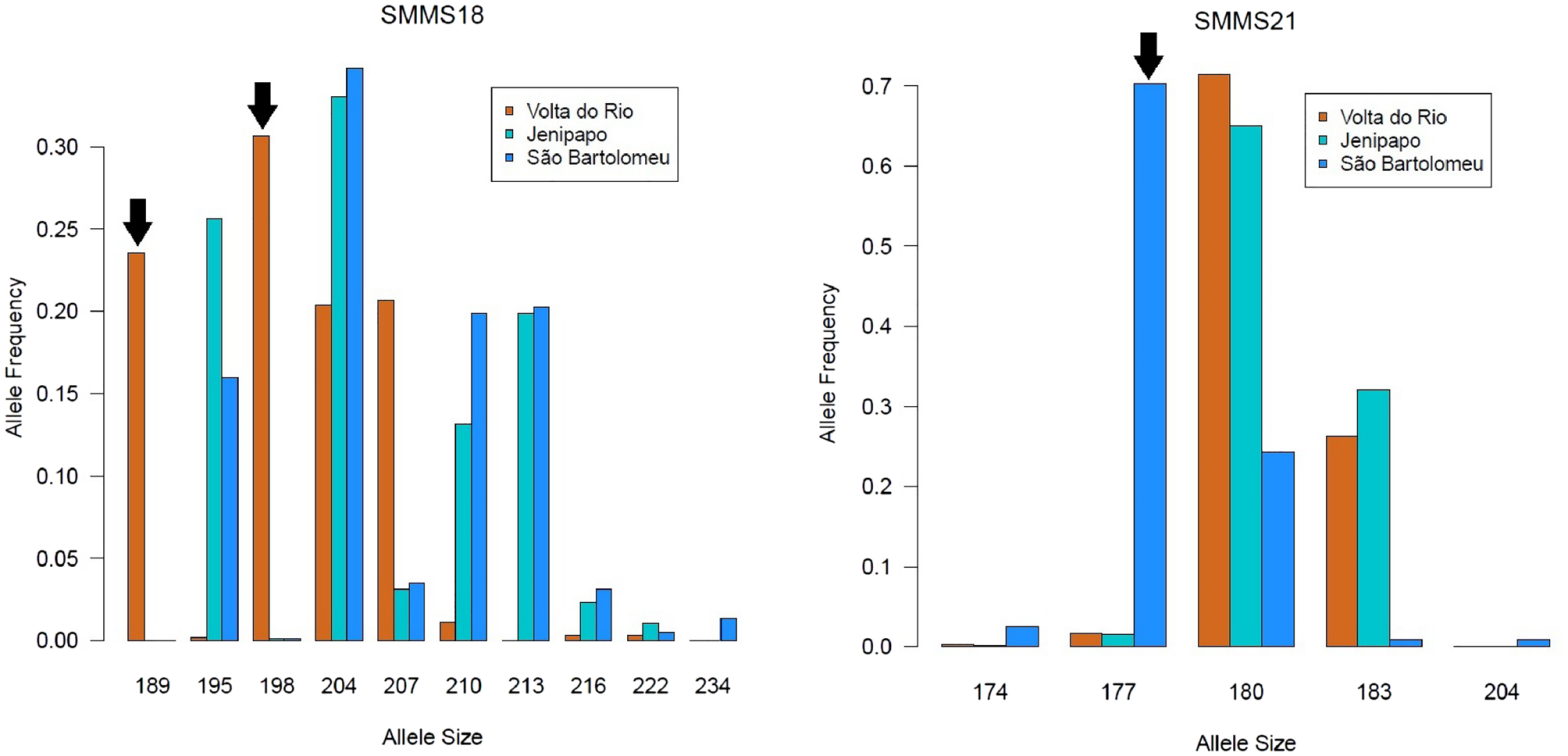
Two examples of loci with alleles that reach a high frequency in one community while being rare or absent in the other communities. Arrows point to the diagnostic markers. At SMMS18 the 189 and 198 base-pair alleles are clear markers of infrapopulations sampled in the Volta do Rio community. The 177 base-pair allele at SMMS21 is a clear marker of infrapopulations sampled in the São Bartolomeu community.

**Figure 2:**
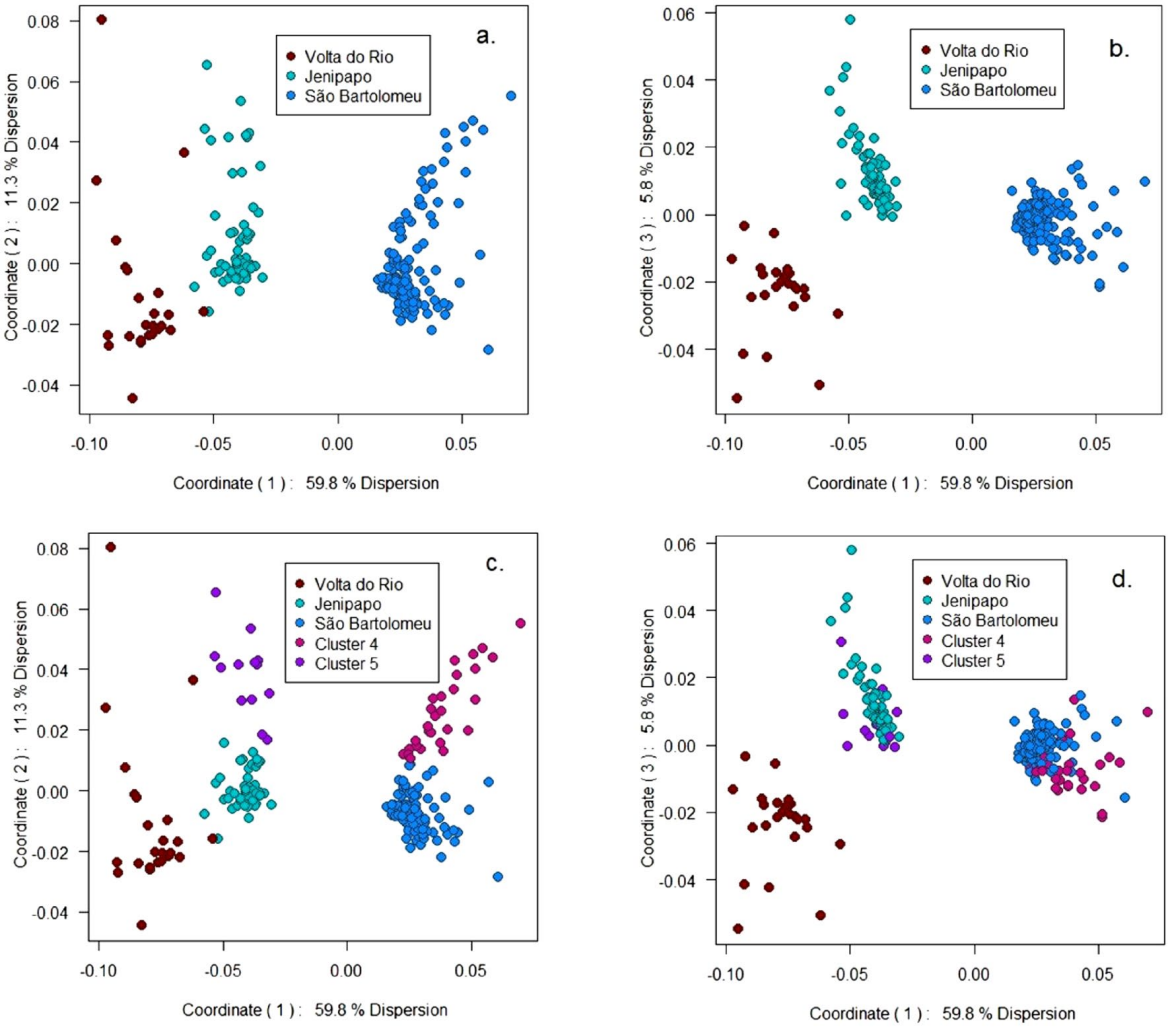
Principal Coordinates of Genetic Distance Matrix. (**a**) PC1 versus PC2 with infrapopulations color coded according to community sampled. (**b**) PC1 versus PC3 with the same coding as in (**a**). (**c**) PC1 versus PC2 with infrapopulations color coded according to cluster analysis results with $K=5$. (**d**) PC1 versus PC3 with the same coding as in (**c**).

**Figure 3:**
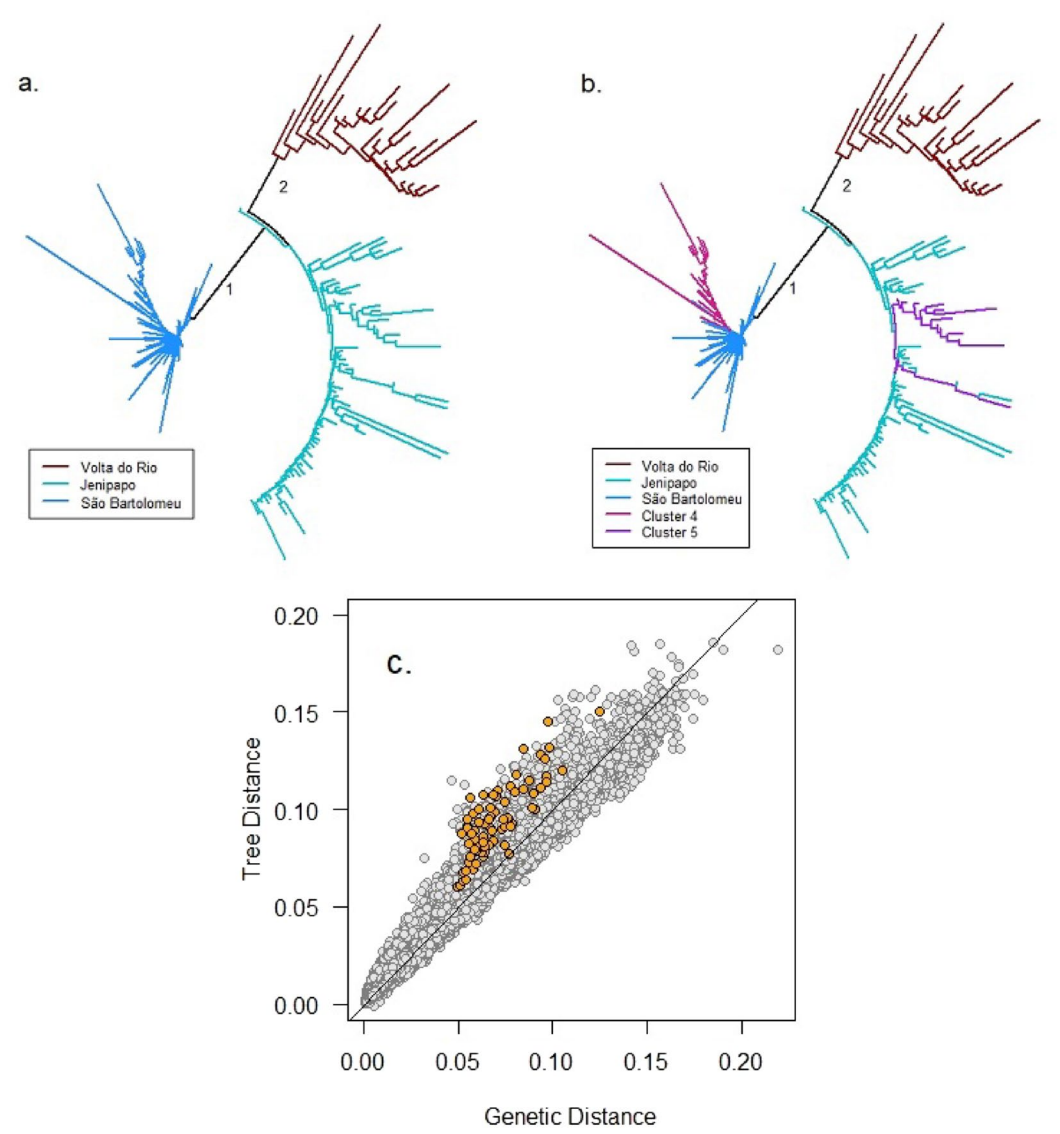
(**a**) Fan plot of the neighbor-joining tree with tips labeled according to communities. (**b**) Fan plot of the neighbor-joining tree with tips labeled according to results for K-means cluster analysis with $K=5$. (**c**) Genetic distances computed as the sum of branch lengths on the neighbor-joining tree plotted against genetic distances computed directly from allele frequencies. The orange-filled points indicate comparisons between infrapopulations in Cluster 4 with those in Cluster 5.

**Figure 4:**
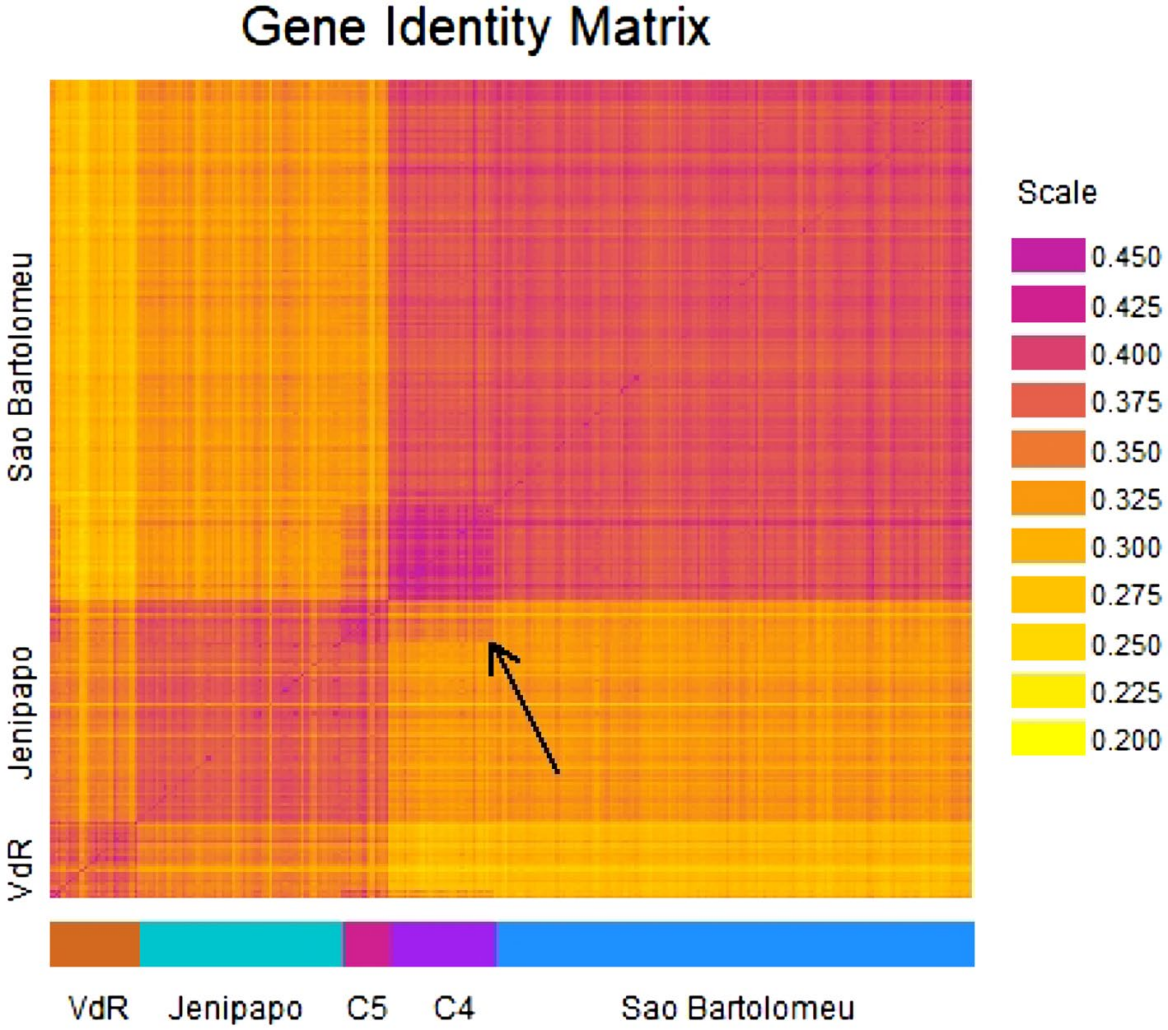
Heatmap of Gene Identity Matrix. Each ‘pixel’ on the diagonal corresponds to the gene identity within an infrapopulation. Each ‘pixel’ on the off-diagonal corresponds to the gene identity between two infrapopulations. Rows and columns are ordered using the results from K-means clustering with K = 5. VdR = Volta do Rio, C4 is a subgroup of infrapopulations sampled in São Bartolomeu and C5 is a subgroup of infrapopulations sampled in Jenipapo. The arrow identifies a block of increased gene identity between infrapopulations in C4 and C5.

## Results

The 14 microsatellite loci that we analyze here are highly polymorphic in the three Brazilian communities. The number of alleles per locus ranges from 4 to 15 with a median of 10.14 (Table 1). The allele frequencies at most loci are similar in the three communities. However, a few loci have alleles that reach a high frequency (*e.g.,* $p \ge 0.20$) in one community while being absent or having a low frequency in other the other communities (Fig. 1). Such alleles are diagnostic markers of the communities in which they are found. Notably, Volta do Rio harbors common alleles at the loci SMMS18 (Alleles 189 and 198), 1F8A (Alleles 147 and 151), and 13TAGA (Allele 102) that are rare or absent in both São Bartolomeu and Jenipapo. São Bartolomeu harbors alleles at the loci SMMS21 (Allele 177) and SM13-410 (Allele 188) that are rare or absent in both Jenipapo or Volta do Rio. We did not find diagnostic alleles for infrapopulations sampled in Jenipapo. The Online Supplemental Information presents the allele frequency profiles for each of the remaining twelve loci.

Nei’s gene identity coefficients^17,18^ vary greatly by infrapopulation and by locus. Table 1 shows gene identity coefficients for the loci individually at three levels of population structure: allele frequencies within infrapopulations ($J_S$), allele frequencies within communities ($J_C$), and allele frequencies in the total population of three communities ($J_T$). The locus-specific gene identity for the total population ($J_T$) ranges from 0.147 to 0.677, with an average of 0.302. For communities ($J_C$), the locus specific gene identities increase with a range of 0.152–0.678 with an average of 0.331. The locus-specific gene identity of infrapopulations ($J_S$) within communities is the highest with a range of 0.169–0.696 with an average of 0.352.

Focusing on gene identity in the individual infrapopulations reveals substantial variation, even for infrapopulations in the same community. The range of gene identity among infrapopulations in São Bartolomeu is [0.321–0.493] with a mean of 0.352. In Jenipapo, the range of gene identity among infrapopulations is [0.307–0.441] with a mean of 0.348. The range of gene identity among infrapopulations in Volta do Rio, is [0.301–0.443] with a mean of 0.357. These community averages (0.352, 0.348, and 0.357) are surprisingly similar despite the differences among infrapopulations within communities (see Online Supplement for full distributions).

Nei’s genetic differentiation coefficients (*i.e., G-statistics)* are quite variable across loci. However, the multiple locus averages show that allele frequencies are significantly different at the level of infrapopulations within the same community $G_{SC} = 0.032$, at the level of allele frequencies in communities relative to the total population $G_{CT} = 0.041$, and at the level of infrapopulations relative to the total population $G_{ST} = 0.072$. The 95% bootstrap confidence intervals show that each coefficient is statistically significant (Table 1). The *G-statistic* values are notably higher for the loci that harbor the diagnostic alleles.

Figure 2 presents plots of the first three principal coordinates (PC) of the distance matrix. PC 1 separates the infrapopulations in São Bartolomeu from those in Jenipapo and Volta do Rio. Additionally, it separates most infrapopulations in Jenipapo from infrapopulations in Volta do Rio. However, there is a small overlap of samples from Jenipapo and Volta do Rio on PC1. The communities do not separate on PC2 (Fig. 2a). Rather than distinguishing among communities, PC2 appears to differentiate infrapopulations within communities. It is unexpected that genetic differences within different communities would segregate on the same principal coordinate. PC3 distinguishes between infrapopulations in Jenipapo and Volta do Rio. The combination of PC1 and PC3 completely separates the infrapopulations in all three communities (Fig. 2b).

Figure 3a displays the unrooted neighbor-joining tree computed from Nei’s minimum genetic distances with the tips color-coded by sampled community. Three distinct clusters appear. Each cluster corresponds to one of the communities sampled. However, the samples from Volta do Rio emerge from within the Jenipapo cluster. Thus, the first branch that includes all infrapopulations sampled within Jenipapo also includes all infrapopulations sampled in Volta do Rio. This is consistent with the fact that the locations of samples from Jenipapo and Volta do Rio on PC1 and PC3 show some overlap.

The K-means cluster algorithm with $K=3$ partitioned the infrapopulations into groups that perfectly matched the sampled communities, *i.e.,* all members of Jenipapo belonged to one cluster, no infrapopulation from another communitity was assigned to the cluster containing Jenipapo, etc. Thus, the color coding of infrapopulations in Figs. 2a,b and 3a,b indicate the cluster analysis results in addition to the sampling locations.

To search for further levels of genetic structure we applied K-means cluster analysis with $K>3$. We found stable solutions with $K=4$ and $K=5$. However, with $K>5$ the method produced unstable results in the sense that clusters found on replicate runs with the same *K* did not match each other in a one-to-one fashion (see Methods). Cluster analysis with $K=4$ maintains the Jenipapo and Volta do Rio clusters that were produced by $K=3$, but São Bartolomeu divides into a main group of $N=132$ infrapopulations and a subgroup of $N=29$ infrapopulations. Hereafter, we will refer to this subgroup as Cluster 4. Cluster analysis with $K=5$ maintains Volta do Rio cluster and the division of São Bartolomeu produced by $K=4$, but it now divides infrapopulations in Jenipapo into a main group of $N=56$ infrapopulations and a subgroup of $N=13$ infrapopulations. Hereafter, we will refer to this subgroup as Cluster 5. Figure 2c,d reproduce the principal coordinate plots and Fig. 3b reproduces the neighbor-joining tree, this time with labels that show the clusters that emerged with $K=4$ and $K=5$. Principal coordinate 2 is now seen to distinguish Cluster 4 from the other infrapopulations in São Bartolomeu, and likewise to distinguish Cluster 5 from the other infrapopulations in Jenipapo. The neighbor-joining tree further demonstrates the distinctiveness of Clusters 4 and 5. The 29 infrapopulations in Cluster 4 join together on a branch that includes only 3 infrapopulations from the main São Bartolomeu cluster. Similarly, 12 of the 13 infrapopulations in Cluster 5 join together before joining with other infrapopulations in Jenipapo..

The fact that one coordinate (PC2) reveals both Cluster 4 and Cluster 5 is unexpected. The reason for this is that the vast majority of members of Cluster 4 are concentrated on the neighbor joining tree as descendants of a single branch that emerges from São Bartolomeu, while most members of Cluster 5 are concentrated on a single branch that emerges from Jenipapo. Thus, the neighbor joining tree gives the appearance that these two clusters are independently evolving lineages. We take two steps to evaluate the possibility that Cluster 4 and Cluster 5 have a stronger genetic relationship than the neighbor joining tree reveals.

The first step in our evaluation is to compare the observed genetic distances with genetic distances derived from the neighbor-joining tree. We have computed the latter distances by taking the sum of all branch lengths connecting a pair of infrapopulations. Random deviations from a tree-like structure will produce both positive and negative differences between the observed genetic distances and the tree distances. It is note worthy here that the tree distances are larger than the observed genetic distances for all 377 pairs of infrapopulations from Cluster 4 with Cluster 5. In other words, the tree over-estimates genetic distances in comparisons between a member of Cluster 4 and a member of Cluster 5 (Fig. 3c).

The second step in our evaluation of the relatedness of infrapopulations in Cluster 4 with those in Cluster 5 was to plot a heatmap of the gene identity matrix with infrapopulations arranged in the specific order Volta do Rio, Jenipapo, Cluster 5, Cluster 4, and São Bartolomeu, as suggested by the neighbor-joining tree (Fig. 4). It is easy to verify the main results from the cluster analysis, neighbor joining tree, and principal coordinates in the heatmap. The infrapopulations from each community form a block of increased gene identity (as indicated by darker shading). The gene identity between infrapopulatlions in different communities is typically lower than the gene identity between infrapopulations in the same community. It is noteworthy that gene identity between infrapopulations in Volta do Rio and infrapopulations in Jenipapo is higher than is the gene identity between infrapopulations in either of these communities and infrapopulations in São Bartolomeu. The infapopulations in Cluster 4 form their own block, as do those in Cluster 5. We can also see that the gene identity between infrapopulations in Cluster 4 and infrapopulations in Cluster 5 is higher than the gene identity between typical infrapopulations in São Bartolomeu.

## Discussion

Schistosomiasis persists throughout the world and is now spreading from rural to urban areas despite control efforts, including successful treatment using the drug praziquantel^6,19^. Methods to detect the sources of recurring infections and the spread of new infections are of paramount importance, and genetic analysis has become prominent in the epidemiological toolbox^9^. In this study, we analyze allele frequency profiles calculated from fourteen microsatellite loci in *Schistosoma mansoni* infrapopulations existing in three communities in the state of Bahia, Brazil. Two of the communities are located in close proximity to each other in a rural environment, whereas the third community is located distantly in an urban neighborhood. We were interested in three questions. First, do the infrapopulations in different communities form distinctive gene pools that might enable tracing the origin of infections? Second, are there subgroups within communities that might inform on transmission patterns at a very local level? Third, are infrapopulations in different communities differentially related?

We found a one-to-one correspondence between genetic clusters found by K-means cluster analysis and communities when $K = 3$. These clusters are also easily identified in a neighbor-joining tree and principal coordinates plots. K-means analysis with $K > 3$ also reveals genetic clusters of parasites at the sub-community level. A surprising finding is a genetic relationship between subgroups in widely separated human communities. This connection suggests the existence of common transmission sites that have wide influence.

We have estimated allele frequency profiles in pooled DNA from eggs in stool samples from individual hosts. We assayed only tri- and tetranucleotide repeat loci to avoid stutter peaks that obscure the analysis of dinucleotide repeat loci. By contrast to pooled DNA samples, it is possible to hatch miricidia from single eggs and thereby obtain individual genotypes for single nucleotide polymporphisms. The single egg approach enables the estimation of a finer level of population structure, *i.e*. inbreeding and individual ancestry, but it has the drawback of limiting the sample size from each infrapopulation, and ultimately the number of infrapopulations from a locality. Our pooled DNA approach yields large samples, which are beneficial for accurately estimating differentiation and diversity parameters. These parameters are of high interest for public health and tracing the spread of infections.

We calculated Nei’s gene identity coefficients at different levels of population strucutre. The most basic unit in our analysis is the infrapopulation, which consists of all the worms inhabiting a single person^10^. We estimated allele frequencies for the infrapopulation within each sampled person from the pooled DNA extracted from all eggs excreted in a fecal sample. Studies have shown that the number of worms in an infrapopulation varies greatly from a few reproductive pairs to hundreds of pairs with an average in the forties. Gene identity varies substantially among infrapopulations in the same community, although the average gene identity in the three communities is roughly the same, 0.348–0.357. Worm burden is a principal determinant of gene identity in infrapopulations, although there are contributing factors such as inbreeding and genetic kinship among the worms within an infrapopulation^8,9,13^. The high variability of gene identity among infrapopulations within a community is important because migration of *S. mansoni* to new locations involves human feces which transfer fertilized eggs from an entire infrapopulation. It is possible that a single migration event transfers considerable genetic diversity, and might be sufficient to found a component population in a new location.

We used gene identity estimates to calculate Nei’s gene differentiation statistics (*G-statistics*) at three levels of population structure. Our *G-statistics* are based on the multiple locus averages (Table 1), which is typically necessary to achieve precise estimates. The values, $G_{SC} = 0.032$ and $G_{ST} = 0.072$, show that infrapopulations were diverged from each other relative to both their community averages and the suprapopulation composed of all three communities. The value of $G_{CT} = 0.032$ shows that the infrapopulations are also differentiated from each other at the community level. The K-means cluster algorithm with $K=3$ partitioned our total set of 254 infrapopulations into groups that perfectly matched the three communities. The cohesiveness of these genetic clusters is visually apparent on the principal coordinates plots (Fig. 2). In addition, a neighbor-joining tree produces three main clusters of infrapopulations. This representation of the data presents one of the rural populations, Volta do Rio, as a subgroup of the other, Jenipapo.

A limitation of the three-level hierarchy used in *G-statistic* analysis and similar methods such as Wright’s *F-statistics*^20^ and Jost’s *D-statistics*^21^ is that they force the genetic structure of the population onto the sampling units defined by the investigator. These units are usually operationally defined and may, or may not, correspond to natural divisions in a species^22^. Indeed, it is possible that unrecognized groupings may exist within sampling units such as communities, and that there may be complex patterns of relationship across sampling units. To investigate these possibilities we applied K-means cluster analysis with $K>3$. As noted, $K=4$ divides the São Bartolomeu sample into two clusters, and $K=5$ divides the Jenipapo sample into two clusters while maintaining the partition of São Bartolomeu produced by $K=4$. The clustering with $K=5$ resolves an enigma observed in the principal coordinates analysis, namely that coordinate 2 does not resolve the sampled communities, whereas this is achieved by the less important coordinate 3 (Fig. 2a,b). With the points labeled according to the $K=5$ result, it is now clear that coordinate 2 differentiates Cluster 4 from the remainder of infrapopulations in São Bartolomeu and it differentiates Cluster 5 from the remainder of infrapopulations in Jenipapo (Fig. 2c,d). However, this introduces a new puzzle. To be differentiated by the same axis, the allele frequency profiles in Clusters 4 and 5 must be correlated with each other. However, a correlation of this nature is incompatible with a pure tree-like data structure. Figure 3c shows that the neighbor-joining tree, which places Cluster 4 within São Bartolomeu infrapopulations and Cluster 5 within Jenipapo infrapopulations, over-estimates all distances between the infrapopulations in Cluster 4 compared with infrapopulations in Cluster 5. Figure 4 shows the reticulate pattern most clearly. Members of Cluster 4 have high gene identity with infrapopulations in São Bartolomeu and members of Cluster 5 have high gene identity with infrapopulations in Jenipapo, but a box of high gene identity encloses Cluster 4 with Cluster 5.

Admixture will cause the nonindependence of evolutionary lineages. In terms of Cluster 4 in São Bartolomeu and Cluster 5 in Jenipapo, there are several possible scenarios for infrapopulation mixing. First, an infrapopulation from São Bartolomeu was deposited in Jenipapo. Second, an infrapopulation from Jenipapo was deposited in São Bartolomeu. Third, an outside source deposited the same infrapopulation into members of both the São Bartolomeu and Jenipapo communities. This could happen in either of two ways, (a) an infected outsider visited both communities, or (b) members of both communities traveled to the same location, acquired their infections, and then returned home. We find that the first two scenarios are less compatible with the pattern in the gene identity matrix than the third scenario. Our reasoning is as follows, suppose that a randomly chosen subset of infrapopulations in São Bartolomeu were to reproduce in Jenipapo, then we would see increased gene identity between those infrapopulations in São Bartolomeu and their descendants in Jenipapo, but the donor infrapopulations would not stand out as a unique cluster in São Bartolomeu. We would not see Cluster 4 appear as a dark block in the matrix. The second scenario would produce the reciprocal pattern. There would be increased gene identity between the donor lineages in Jenipapo and their descendants in São Bartolomeu, but not a distinct block within Jenipapo. Finally, the third scenario has more potential to create subclusters of infrapopulations with high gene identity within sampled communities and high gene identity between subclusters in different communities. This is the pattern of gene identity displayed by the matrix. Importantly, the geographic separation of São Bartolomeu and Jenipapo (200 km) requires that the humans who transferred the parasites traveled over a great distance. However, our data do not allow us to distinguish between possibilities (a) and (b) noted above.

We wish to emphasize that our reasoning in favor of the scenario involving outside migration is heuristic, as we have not rejected any migration scenario using a formal statistical test. Nevertheless, there are several other hints that unsampled genetically diverse schistosome populations have influenced the allele frequency profiles in the three communities that we have sampled. First, the distribution of diagnostic alleles in Volta do Rio and São Bartolomeu is incompatible with gene flow. Even a low level of mixing between these three populations would have been sufficient to place all allelic types in each community. Second, the distribution of diagnositic alleles is also incompatible with a phyletic process. If the branching pattern of the neighbor-joining tree (Fig. 3) represented population fissions and the root of the tree was placed on branch (1), which separates São Bartolomeu from the two rural communities, then the mutations leading to the diagnostic alleles in Volta do Rio would be required to have occurred on branch (2) and risen to high frequency in a short time interval. Alternatively, if the proper root of the neighbor-joining tree was on branch (2), then mutations creating the diagnostic alleles of São Bartolomeu would be required to have occurred on branch (1) and risen to high frequency in a short time interval. Third, the gene identity matrix has a reticulate structure (Fig. 4). Jenipapo forms one box with Volta do Rio and an overlapping box with São Bartolomeu. However, a pure phyletic process would have created perfectly nested boxes^23,24^ and gene flow would have homogenized the frequencies of the diagnostic alleles. We have not yet identified the source of outside migration, but that should be an objective of furture investigations of schistosome populations in Bahia.

In conclusion, the work presented here demonstrates how population genetic studies can reveal unique aspects of schistosomiasis transmission. We found that parasite infrapopulations are genetically differentiated among human communities in Brazil, including those that are in close geographic proximity. In addition, we discovered genetically differentiated parasite subgroups within communities. Taken together, these two findings indicate that residential areas serve to genetically isolate schistosome populations. However, we have also found strong evidence that the overall pattern of isolation is incomplete and broken by occasional long-range migration. Such long-range migration must be human mediated because the snail hosts are highly sedentary. The migration of schistosomes from one location to another does not require that infected humans change their residences. It is possible that itinerant activities, either professional or recreational, are responsible for the transfer of schistosomes. A surprising finding is the genetic relationship between subgroups in widely separated human communities. This suggests the existence of transmission centers that have wide influence. At the very least, our work shows that permanent eradication of schistosomes in particular communities will require both the identification of regional infection reservoirs and intensive action within communities. While the microsatellite data that we have used supports the present findings and conlclusions, we note that full genome data may be helpful in future analyses that focus on the fine details of Schistosome population structure and transmission dynamics.

## Methods

### Sample collection and human subjects approval

The focus of this study is *Schistosma mansoni* carried by infected residents of three communities located in the State of Bahia, Brazil. The first community is the urban neighborhood of São Bartolomeu, which is located in the City of Salvador. The population of São Bartolomeu is approximately 6,000 people. Some 1,423 residents of São Bartolmeu over 4 years of age and living closest to the Cobre River were contacted. Each responded to a demographic questionnaire and parasitologic examinations. The second and third communities, Jenipapo and Volta do Rio, are rural communities located 200 km southwest from Salvador in the municipality of Ubara. The populations of Jenipapo and Volta do Rio were 482 and 367, respectively. All inhabitants over 1 year of age in these communities were invited to participate and a parasitologic examination was administered to those who agreed to particiapte. Fecal samples were processed by the Kato-Katz method and examined microscopically to identify ova of *S. mansoni*. Each stool positive for *S. mansoni* ova was homogenized, and DNA was extracted using a standard phenol-chloroform extraction procedure followed by treatment with hexadecyltrimethylammonium bromide (CTAB) to remove PCR inhibitors. DNA from fecal samples was analyzed for N = 219 infrapopualtions in São Bartolomeu, N = 80 in Jenipapo, and N = 38 in Volta do Rio.

Following PCR amplification and capillary electrophoresis, allele frequency profiles for 14 microsatellite loci were constructed for each fecal sample. The microsatellite loci are a subset of a panel containing loci with tri- or tetra-nucleotide repeat motifs. The microsatellite panel and laboratory procedures were described recently^16^. The 14 loci used in this study were SMMS2, SMMS13, SMDA23, SMU31768, 15J15A, 29E6A, SM13-478, SMMS3, SMMS18, 1F8A, 13TAGA, SMMS16, SMMS21, and SM13-410.

As described in earlier publications, both data collections were approved by The Committee on Ethics in Research of the Oswaldo Cruz Foundation of Salvador, Bahia, the Brazilian National Committee on Ethics in Research and the Institutional Review Board for Human Investigation of University Hospitals Case Medical Center, Cleveland, Ohio. All subjects provided written informed consent, or in the case of minors, written consent was secured from their guardians. All aspects of the study were conducted according to the principles expressed in the Declaration of Helsinki^5,15^.

### Data analysis

Each DNA sample from an infected person represents an infrapopulation of worms. Our primary unit of analysis is the 14 locus allele frequency profile from each infrapopulation. Complete data is required for one of our principal statistical methods (K-Means clustering). Therefore, we analyzed only those infrapopulations for which laboratory assays were successful at all 14 loci. While some of our other methods accommodate missing data, we prefer to perform all statistical analyses using the same data. Thus, the numbers of infrapopulations analyzed were N = 161 for São Bartolomeu, N = 69 for Jenipapo, and N = 24 for Volta do Rio.

We used Nei’s gene identity statistic as the first step to summarize patterns of genetic diversity within and between infrapopulation allele frequency profiles^17,18^. For a single genetic locus with allele frequencies measured for a collection of population subdivisions, the gene identity within the *i*th subdivision is estimated as $J_i=\sum _k p^2_{ik}$, where $p_{ik}$ is the frequency of the *k*th allele in subdivision *i*. The summation is taken over all alleles at the locus. We calculated gene identity at three levels of population structure, $J_S$ for gene identity within infrapopulations, $J_C$ for gene identity within communities, and $J_T$ for gene identity within the total collection from all communities. To measure genetic differentiation at different levels of community structure, we use Nei’s coefficients of gene differentiation, which are familiarly known as *G-statistics*^18^. We compute $G_{SC} = (J_S-J_C)/(1-J_C)$ to measure diversity within infrapopulations relative to their communities, $G_{CT} = (J_C-J_T)/(1-J_T)$ to measure diversity within commnities relative to the total population, and $G_{ST} = (J_S-J_T)/(1-J_T)$ to measure diversity within infrapopulations relative to the total population. After calculating the gene identity and genetic differentiation statistics individually for each locus, we use the mutliple locus averages for further analyses. Hereafter, we use the symbols *J* to represent the average of gene identities taken over all 14 loci. We used the bootstrap method to calculate 95% confidence intervals for the multiple locus estimates of gene identity and genetic differentiation coefficients^25^.

We use the gene identity between infrapopulations to reveal fine-scale relationships. For a single locus, the gene identity between the $i^{th}$ and $j^{th}$ subdivision is estimated as $J_{ij}=\sum _k p_{ik}p_{jk}$. As before, we average gene identities between populations over the 14 loci. Cavalli-Sforza and Piazza^23,26^ and others^24^ have shown that a matrix of gene identities, with elements $J_i$ placed on the diagonal and elements $J_{ij}$ placed on the corresponding off-diagonals will take a simple block-like structure if related populations are ordered adjacent to each other in the matrix. We use a heat-map to examine the gene identity matrix for block-like patterns that indicate population relationships.

We convert the gene identitfy matrix into a matrix of Nei’s minimum genetic distance to quantify the differentiation between population subdivisions. The minimum genetic distance between the *i*th and *j*th subdivisions for a single genetic locus is computed as $D_{ij} = \frac{1}{2} \sum _k (p_{ik}-p_{jk})^2 =(J_i + J_j)/2 - J_{ij}$. As before, we use multiple locus averages of gene identity to compute genetic distances. To visualize the genetic distances among subpopoulations along a set of uncorrelated axes, we compute the principal coordinates of the distance matrix. A distance matrix will have many principal axes. However, the first few axes typically account for a substantial portion of the total dispersion in the matrix and present major patterns^27^. Principal coordinates calculated from Euclidean distances have been shown to be equivalent to principal components of the original data matrix^28^.

We take two approaches that are independent of sampled communities to find clusters of infrapopulations that might reveal component population structure. First, we use K-means clustering to partition our data into a pre-specified number of groups (*K*). We evaluated models with $(2 \le K \le 8)$. We used the R function *kmeans* with the arguments iter.max = 20 and nstart = 50. These values are more stringent than the default values of iter.max = 10 and nstart = 1 and should insure that the numerical solutions are close to the optimum. We repeated analyses using these parameters of each *K* ten times and checked to see if the cluster partitions matched one-to-one. With $K\ge 6$ the additional clusters contained small numbers of individuals and were not replicated on repeated runs. Therefore, we present the results for three models: $K = 3$, $K = 4$, and $K = 5$. We were particularly interested in whether or not the $K=3$ cluster model would sort infrapopulations into groups that reproduced the three communities sampled. We were also interested in whether or not higher levels of *K* would detect substructure within the three sampling locations. Second, we compute an unrooted neighbor-joining tree from the minimum genetic distance matrix^29^. Once again, we were interested in whether or not the samples from the three communities would appear as distinct branches on the neighbor-joining tree. K-means and neighbor-joining take opposite approaches to clustering. Whereas, K-means is a divisive method that starts with the entire data set and divides it into K-subgroups, neighbor-joining is an agglomerative method that successively fuses pairs of groups until the entire data set is contained in one cluster. We were interested to see if these opposite approaches would find the same clustering structure within the entire set of 254 infrapopulations.

We performed all data analyses using the R language and statistical computing environment. We wrote original functions for data manipulations and to calculate Nei’s genetic diversity and genetic distance statistics. We used the ’ape’ package to compute and plot the neighbor-joining tree^30^. All other graphs were produced using R’s base graphics. The cmdscale and kmeans functions in the base package were used to compute principal coordinates and cluster analyses, respectively.

## Supplementary information

Supplementary Figures.
