# Supplementary material for: Cryptic population structure and transmission dynamics uncovered for Schistosoma mansoni populations by genetic analyses
Source: Sci Rep. 2022 Jan 20;12:1059. doi: 10.1038/s41598-022-04776-0 (PMC8776789; doi:10.1038/s41598-022-04776-0)

Supplementary Information for *Cryptic Population Structure and Transmission Dynamics Uncovered for Schistosoma mansoni Populations by Genetic Analyses*.

Jeffrey C. Long, Sarah E. Taylor, Lucio M. Barbosa, Luciano K. Silva, Mitermayer G. Reis, Ronald E. Blanton.

Figure S1. Allele frequency profiles for 12 microsatellite loci not shown in text Figure 1. Three loci harbor high frequency alleles in one component population that are absent or rare in the other two component populations. These alleles are marked by vertical arrows.

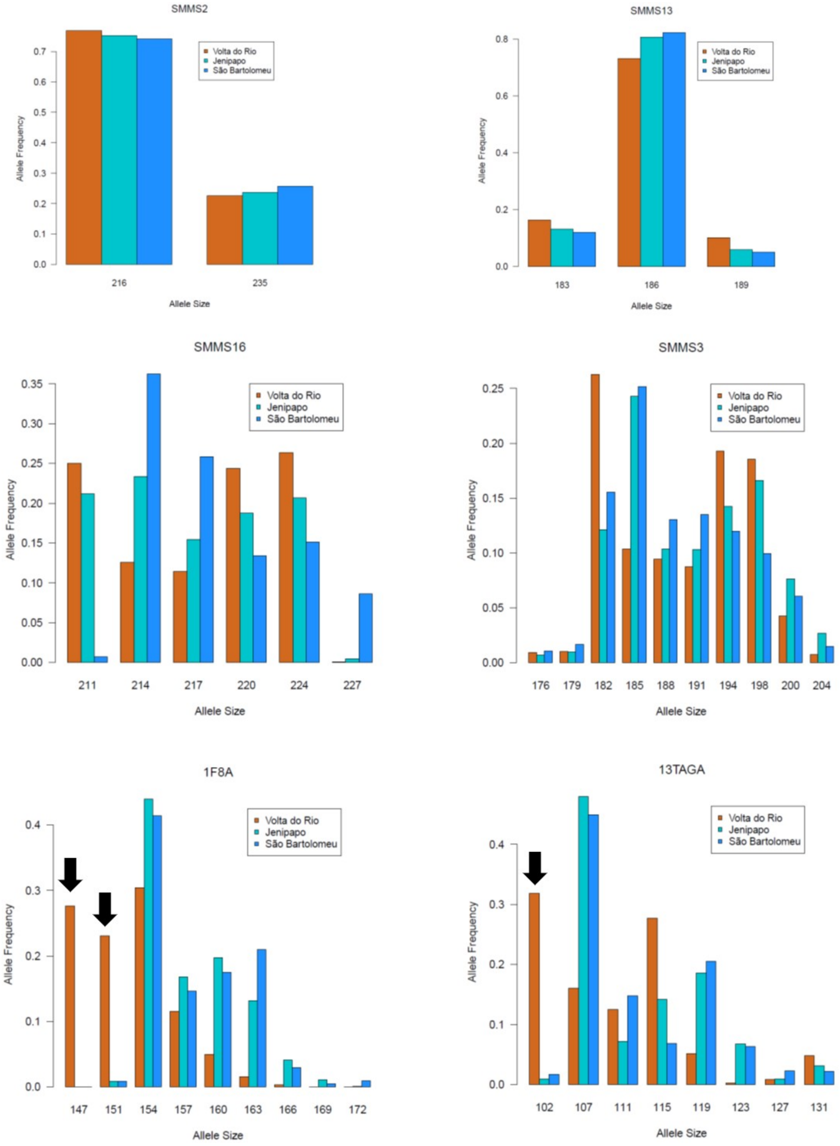

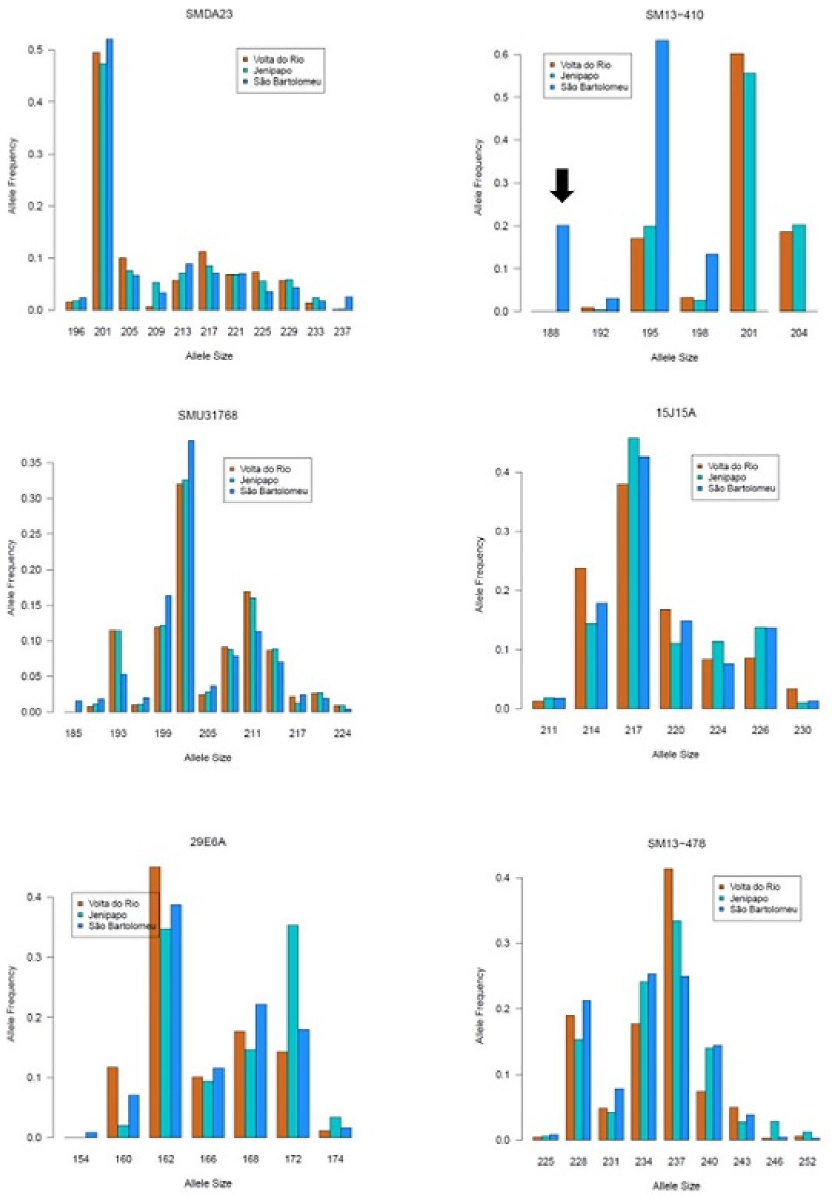
SMDA23
SM13-410
SMU31768
15J15A
29E6A
SM13-478
Volta do Rio
Jenipapo
São Bartolomeu
Allele Frequency
Allele Size

Figure S2. Gene Identity Within Infrapopulations displayed separately for the component populations São Bartolomeu, Jenipapo, and Volta do Rio.

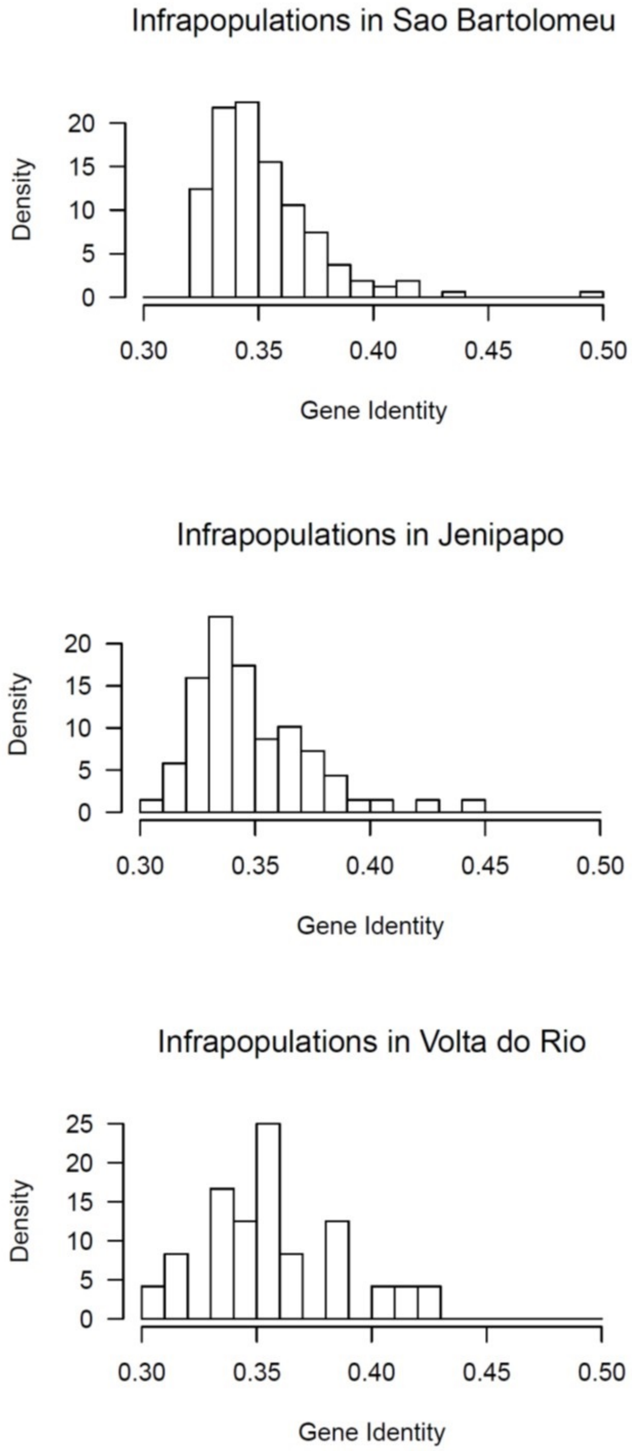

Supplement: Supplementary file 1 — Supplementary Figures. [file 41598_2022_4776_MOESM1_ESM.pdf]
